# Longitudinal changes in disgust sensitivity during pregnancy and the early postpartum period, and the role of recent health problems

**DOI:** 10.1038/s41598-023-31060-6

**Published:** 2023-03-23

**Authors:** Daniela Dlouhá, S. Craig Roberts, Jana Hlaváčová, Kamila Nouzová, Šárka Kaňková

**Affiliations:** 1 Department of Philosophy and History of Science, Faculty of Science, Charles University, Prague, Czech Republic; 2 Division of Psychology, University of Stirling, Stirling, Scotland United Kingdom; 3 ProfiGyn, S.R.O., Municipal Health Centre Prague, Prague, Czech Republic

**Keywords:** Human behaviour, Reproductive biology, Evolution

## Abstract

Disgust is an essential part of the behavioral immune system, protecting the individual from infection. According to the Compensatory Prophylaxis Hypothesis (CPH), disgust sensitivity increases in times of immunosuppression, potentially including pregnancy. We aimed to replicate a previous study observing longitudinal changes in disgust sensitivity in pregnant women. Additionally, for the first time, we explored how recent health problems influence these changes. To do this, we obtained disgust sensitivity measures from 94 women in each trimester and in early postpartum. In contrast to the original study, where disgust sensitivity was highest in the first trimester, we found that overall and animal reminder disgust increased across pregnancy and after birth. In line with the CPH, women who were recently sick in the first trimester had elevated disgust sensitivity at that time. Although disgust sensitivity was significantly higher in the second trimester and postpartum period compared to the first trimester in mothers pregnant with a male fetus, the overall results regarding the effect of fetus sex on disgust sensitivity were mixed. It seems that changing levels of disgust sensitivity during pregnancy and postpartum result from a suite of physiological and psychological changes that occur during this sensitive period of a woman’s life.

## Introduction

The emotion of disgust is thought to act as a defense mechanism against infection by pathogens^1^. Disgust therefore plays a crucial role in the behavioral immune system: by limiting contact with potentially harmful objects, substances or people, it minimizes the risk of infection and related activation of the physical immune system, which is costly to the organism^2^.

Disgust sensitivity, defined by Olatunji et al. in their study^3^ as a predisposition to experiencing disgust towards a wide array of aversive stimuli, varies considerably between individuals. This variation depends partly on age^4,5^, sex^1,6^ and personality traits such as neuroticism^7^ or anxiety^8^. Disgust sensitivity can also change in response to social learning ^9^ or variation in potential pathogen exposure and an individual’s current state of immunity^10^. Indeed, studies have shown that disgust sensitivity is higher in people who are more frequently sick^11^, consider themselves more vulnerable to infection^12^, or who live in a pathogen-rich environment^13,14^. However, other studies do not support the hypothesis, showing no positive association between disgust sensitivity and frequency of sickness during childhood^15^ or being immune-compromised due to medication^16^. Similarly, Tybur et al.^17^ did not confirm the association between recent illness and attention paid to disfigured faces, which was originally observed by Miller and Maner^18^.

Within this literature, a prominent idea regarding the effects of reproductive immunosuppression on disgust sensitivity is the Compensatory Prophylaxis Hypothesis (CPH). According to the CPH, disgust sensitivity should be higher during the luteal phase (usually between the 15th and 28th day of an average cycle) of the menstrual cycle because this compensates for concurrent immunosuppression as the mother’s body prepares for potential implantation^4,19,20^. The underlying mechanism for this compensatory process is thought to be levels of progesterone^19^. In line with this hypothesis, both Fleischman and Fessler^20^ and Żelaźniewicz et al.^21^ found that disgust sensitivity was higher among women with higher levels of progesterone. However, it should be noted that these results were not supported in a more recent study using a longitudinal design^22^.

Shortly after it was first proposed, the CPH was tested further in relation to pregnancy. In a cross-sectional study, Fessler et al.^23^ reported that, compared to later trimesters, women had higher disgust sensitivity in their first trimester of pregnancy. At the time of that study, the first trimester was regarded as a time when the body was in a state of immunosuppression and the authors proposed that disgust sensitivity is elevated to compensate for this. However, this perspective on pregnancy has since been reassessed. Instead of immunosuppression, changes are now viewed as immunomodulation—the immune system is altered to protect the fetus as well as the mother^24^. Nevertheless, the mother’s immune system is still undergoing significant and complex changes during pregnancy, so any increase in disgust sensitivity could still play a significant protective role.

In the intervening years, only two other studies have examined changes in disgust sensitivity during pregnancy. Using a longitudinal design, Żelaźniewicz and Pawłowski^24^ found that women in the first trimester had higher disgust sensitivity on the core subscale of the questionnaire Disgust Scale-Revised (DS-R)^3^, with these scores decreasing in later trimesters and after birth. Women who were pregnant with a boy showed higher disgust sensitivity in the first trimester than women pregnant with a girl, and this difference extended into the second trimester. The authors attributed this to differences in hormonal levels, specifically of testosterone and cortisol, which have been shown to have immunosuppressive effects^25,26^ and to occur at higher levels in women pregnant with a male fetus^27,28^. In another study, Kaňková et al.^29^, focused on disgust sensitivity and the immune system during the first trimester. Disgust sensitivity correlated negatively with cytokine levels such as Eotaxin, IFN-γ, IL-1β, IL-2 IL-4, IL-7, IL-17A, TNF-α, MCP-1, PDGF-BB, and RANTES. In line with the CPH, the authors propose that disgust works as a compensatory system in cases when the immune system of the mother is not sufficiently active.

In this study, we aimed to replicate the design of the previous longitudinal study on pregnant women^30^, measuring disgust sensitivity using the DS-R in each trimester as well as in the postpartum period. Based on the results of the original study, we expected increased disgust sensitivity in the first trimester of pregnancy in comparison with later trimesters and after birth. This effect should be especially prominent in women pregnant with a male fetus. In addition, we explored the potential effect of recent infection-related health problems on disgust sensitivity in each trimester. In line with the CPH, we expected women who had experienced recent health problems to have higher disgust sensitivity than those who had not.

## Methods

### Participants

In total, 106 women without severe chronic diseases or autoimmune disorders were recruited for the study. All were older than 18 and had not conceived by undergoing assisted reproduction. Of this sample, three women did not complete the study and one woman was excluded because of a later-diagnosed twin pregnancy. Eight women miscarried and their data was also excluded from the analysis.

The final sample consisted of 94 women aged between 22 and 43 (Mean = 31.8, SD = 4.48), 52 (55.3%) of whom were primiparous. The majority of the women (70, 74.5%) had completed higher education, while 22 (23.4%) had high school education and two (2.1%) had completed an apprenticeship. Thirty-four (39.0%) women had a monthly household income of 61–91 thousand Czech crowns, 50 (57.5%) had an income between 31 and 60 thousand Czech crowns and three women (3.4%) had an income between 16 and 30 thousand Czech crowns (1000 crowns is equivalent to about US$ 46). Seven women did not answer the question about their income. Most of the sample (68, 73.9%) lived in an area with a population size over 500,000, three (3.3%) in an area with a population size between 50,000 and 500,000, and 21 (22.8%) in an area with fewer than 50,000 (two women did not answer this question). Fifty-one (56.7%) women were pregnant with a boy and 39 (43.3%) with a girl (four women did not give this information). Only two women (2.2%) smoked while pregnant.

### Study procedure

**Figure 1:**
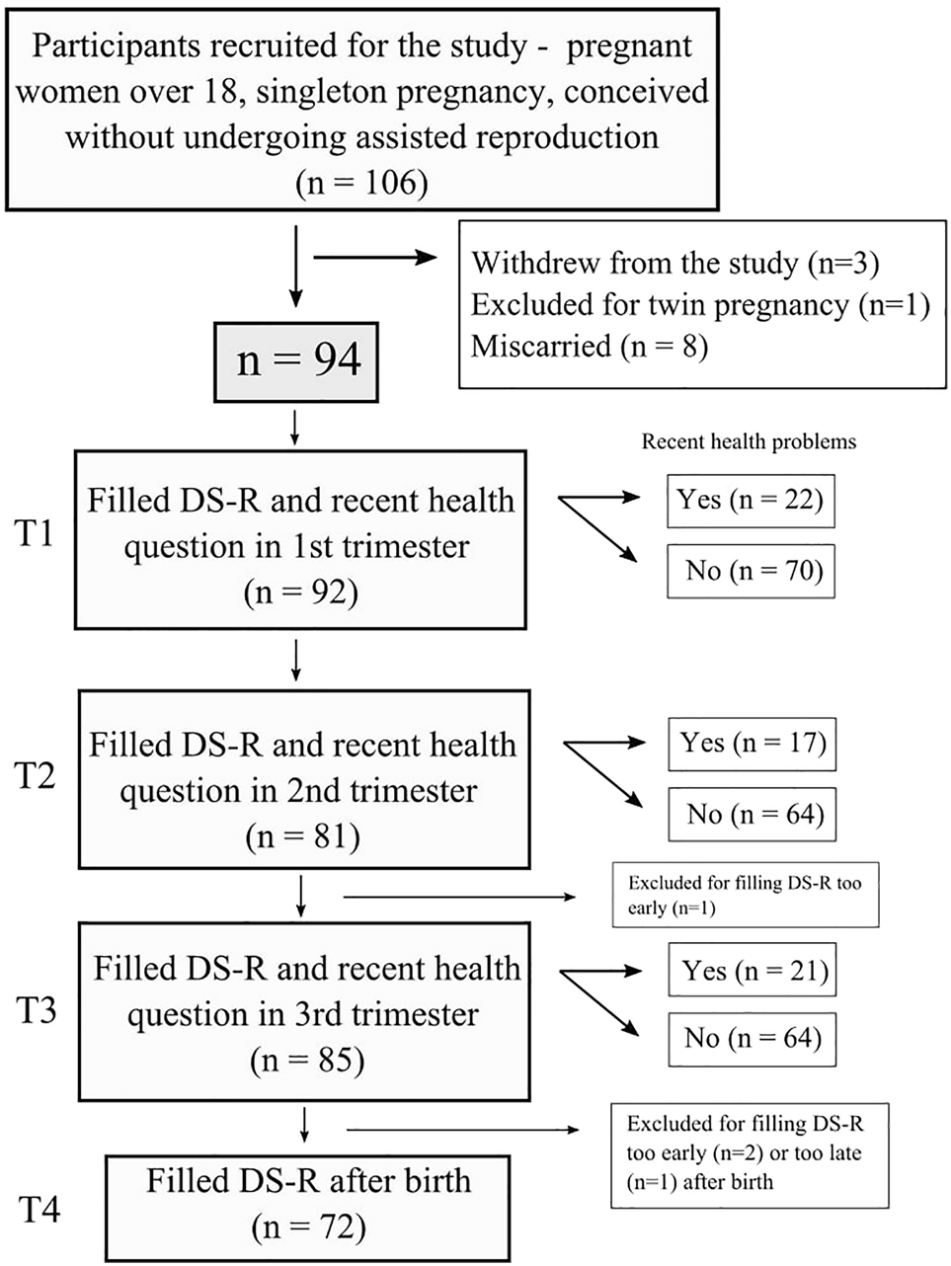
A chart detailing the number of participants in each part of the study.

This study was part of a larger project with the aim of exploring longitudinal changes in pregnancy and their correlations with biological and psychological factors. This project was started in March 2018 in collaboration with a private gynecological clinic, ProfiGyn, in Prague, Czech Republic. The last woman was recruited into the study in July 2019 and the last set of questions during the postpartum checkup was completed in April 2020. The patients of the clinic were invited to participate in the study during their first antenatal medical checkup (T1), between the 4th and 11th week of pregnancy (Mean = 7.30, SD = 1.28). At this time, they completed a background questionnaire (including questions about age, physical parameters, parity, smoking, and a number of demographic questions), a questionnaire measuring disgust sensitivity (Disgust Scale-Revised, DS-R)^3^, and were asked whether they had experienced any health problems in the previous two weeks (yes/no). The DS-R and the recent health question were given to the participants repeatedly throughout pregnancy, in the 2nd trimester (T2) between the 18th and 26th week of pregnancy (Mean = 21.3, SD = 1.15), in the 3rd trimester (T3) between the 28th and 36th week (Mean = 30.1, SD = 1.09), and finally during their postpartum checkup (T4) in the 6–14th week after birth (Mean = 7.33, SD = 1.80). Information about the sex of the baby was obtained from both medical records and questionnaires. Participants who completed the questionnaire too soon in the third trimester (in the 23rd week, n = 1), too soon after birth (specifically in the 3rd week, n = 2), or too late after birth (in the 27th week after birth, n = 1) were excluded from further analysis. The study procedure, including information about the number of completed questionnaires in each trimester, is illustrated in Fig. 1.

Before entering the study, all participants were told that the study focused on changes in pregnancy and their correlations with different biological and psychological factors, as this was the aim of the larger project. The participants were not specifically told about disgust sensitivity, as this could potentially affect their responses. The questions about disgust sensitivity were given to the participants alongside questions about nausea and vomiting in pregnancy. The participants provided written, informed consent and were given an anonymous code to be used in all completed questionnaires. The project was approved by the Institutional Review Board of the Faculty of Science, Charles University (Approval No. 2018/6 and 2019/10). All methods were performed in accordance with the relevant guidelines and regulations.

### Questionnaires

Disgust sensitivity was measured using the Disgust Scale-Revised questionnaire (DS-R)^3^, specifically the Czech version of this questionnaire^31^. This 27-item (25+2) questionnaire is divided into three subscales; (1) core disgust (12 items), focused on pathogen transmission through food and animal or body products, (2) animal reminder disgust (8 items), where the items are focused on injuries or other body envelope violations that could potentially remind people of their own mortality, and (3) contamination disgust (5 items), encompassing possible infection threats through interpersonal contact. The items are scored by the participant on a 5-point Likert scale (0–4). Specifically, in the first set of 14 questions, participants rate statements on a scale ranging from 0 = strongly disagree (very untrue) to 4 = strongly agree (very true). In the second set of 13 questions, the participants rate their disgust to descriptions of different situations on a scale from 0 = not disgusting at all to 4 = very disgusting. Three of the questions are reversed, so the scores must be adjusted accordingly before calculating the final score. The total score ranges from 0 to 100 (0–48 for core disgust, 0–32 for animal reminder disgust and 0–20 for contamination disgust), with higher scores indicating higher disgust sensitivity. If more than 1/5 of each participant’s responses in a subscale was missing, their data were excluded from the analysis. This was done for two participants in the 1st trimester, 13 participants in the 2nd trimester, eight participants in the 3rd trimester, and 20 participants in the six-week postpartum period. If less than 1/5 of the responses were missing for a particular subscale, the missing score was calculated using the participant’s average score of that subscale. This has been done for eight values in the 1st trimester, for three values in the 2nd trimester, for two values in the 3rd trimester and for three values in the postpartum period.

The information about recent health problems included two questions: “Have you had any health problems in the last 14 days?” and “If yes, specify which”. Before the analysis, the details of the reported health problems were reviewed. For the purpose of this analysis, only health problems related to infection were considered as “yes” (such as different symptoms of common cold, bronchitis, nasopharynx infection, tonsilitis, gastric flu), whereas problems associated with pregnancy itself (e.g. back pain) or chronic problems (e.g. allergies, food intolerances) were re-categorized as a “no”. Chronic problems could possibly influence disgust levels differently, perhaps causing a long-term adjustment, which could interfere with our attempt to see changes in disgust levels as a reaction to pathogen contact. All reported health problems and their coding can be found in the Supplement section (Table S1).

### Statistical analyses

Statistical analyses were performed using Jamovi 1.6.16^32^. The normal distribution of the variables was tested using Shapiro-Wilk normality tests. The possible effect of age on disgust sensitivity in each trimester and postpartum period was tested using linear regression. The effect of the gestational age at the time of completing the questionnaires on disgust sensitivity was tested using Kendall correlation for each trimester separately. Effects of binary variables (parity, fetus sex) on disgust sensitivity were tested using t-tests or Mann-Whitney tests (if the assumption of homogeneity was not met).

The longitudinal changes in disgust sensitivity across each trimester, as well as six weeks postpartum, were analyzed using linear mixed models. The repeated measure of disgust sensitivity of each participant was established as the random effect of the model. The covariates of this model were age and the binary variables of parity (primipara/multipara) and fetus sex. These, along with the time of measurement (T1–T4), were established as fixed effects. The interaction between fetus sex and the time of measurement was also added to the model as a fixed effect. In addition to the model, post hoc t-tests were also performed.

The effects of recent health problems on disgust sensitivity in each trimester were analyzed using ANCOVA. The independent variable was the binary variable of recent health problems (yes/no) and the dependent variable was measured disgust sensitivity. Age, parity and gestational age were included as covariates.

Internal consistency was tested for the whole questionnaire, as well as for each individual disgust subscale. The results are reported using a Cronbach’s α value and can be found in the Supplementary material (Table S2). Due to the low internal reliability of the contamination subscale, the results of analyses using this subscale will be reported only for possible comparison with the replicated study, but they will not be discussed. Correlation between the subscales of the DS-R questionnaire was performed and the results can be found in the Supplementary material (Table S3).

## Results

### The effect of age, parity and fetus sex on disgust sensitivity

The linear regressions between age and disgust sensitivity showed a significant effect of age on the core subscale of the DS-R questionnaire in the 1st trimester of pregnancy (t = − 2.28, *p* = 0.025, R^2^ =  0.06), such that older women had lower core disgust scores. There was no significant effect of parity, gestational age or fetus sex on disgust sensitivity. There was a small but non-significant effect (*p* = 0.084, Cohen's d  =  -0.38) of fetus sex on the core subscale in the 3rd trimester, such that women pregnant with a male fetus had higher disgust sensitivity. For more details of analyses, see Table S4 in the Supplement section. Based on these results, age was established as a covariate in all further analyses, as well as fetus sex, mostly because of its significance in the replicated study.

### Longitudinal changes in disgust sensitivity

The linear mixed model included repeated measurements of disgust of each participant as the random effect and age and fetus sex as covariates. The covariates, along with the time of measurement (T1-T4, here referred to as period) were the fixed effects of the model. Since the original study^30^ showed a significant effect of fetus sex, and we observed a small, though non-significant, effect of sex on the core disgust subscale in T3, the interaction of sex and the period (T1-T4) was added to the model. The results showed significant longitudinal changes in the overall DS-R score (F = 3.06, *p* = 0.029, R^2^ marginal = 0.02) and the animal reminder subscale (F = 5.97, *p* < 0.001, R^2^ marginal = 0.02), but not the core subscale (F = 0.36, *p* = 0.783, R^2^ marginal = 0.03) or the contamination subscale (F = 1.93, *p* = 0.125, R^2^ marginal = 0.01). The effects of maternal age and fetus sex on all DS-R scores were not significant and neither was the effect of the interaction of sex and the period. For detailed results, see Table 1.

**Table 1:** The effects of fixed factors and covariates on disgust sensitivity changes; results of the linear mixed model.

	Overall DS-R			Core			Animal reminder			Contamination		
	df	F	*p*	df	F	*p*	df	F	*p*	df	F	*p*
Age	1,89.3	0.41	0.522	1,89.2	1.12	0.293	1,89.1	0.37	0.543	1,89.5	0.21	0.646
Sex	1,89.3	0.66	0.418	1,89.3	1.49	0.225	1,89.2	0.34	0.560	1,89.5	< 0.01	0.993
Period	3,234.4	3.06	**0.029**	3,235.1	0.36	0.783	3,234.4	5.97	**< 0.001**	3,235.2	1.93	0.125
Sex * Period	3,234.4	2.33	0.075	3,235.0	1.40	0.244	3,234.3	2.05	0.108	3,235.2	0.66	0.577

The variable “sex” refers to the sex of the fetus and the variable “period” refers to the time of measurement—in the 1st (T1), 2nd (T2), or 3rd (T3) trimester of pregnancy or after birth (T4).

Significant values are in [bold].

**Figure 2:**
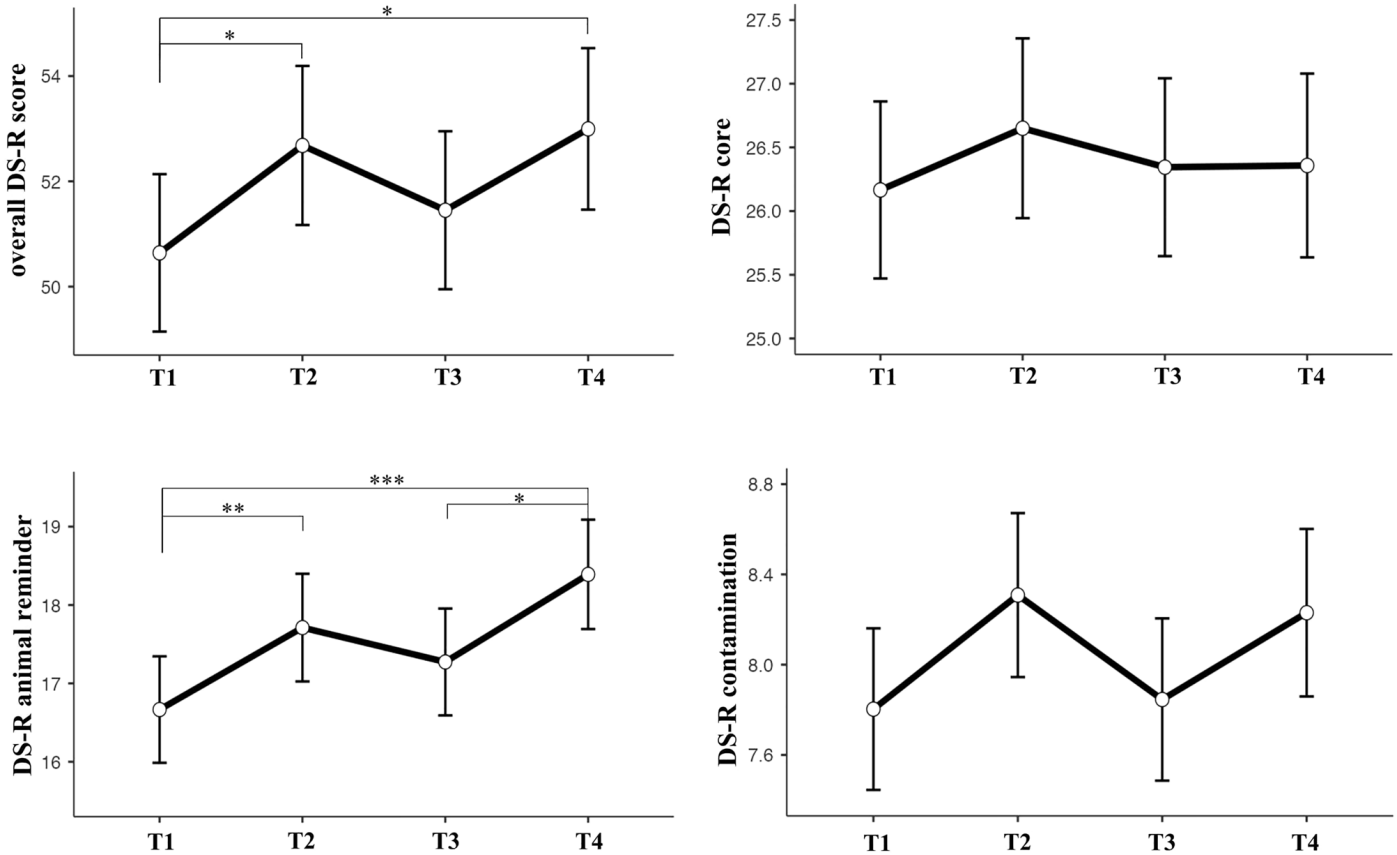
Longitudinal changes during pregnancy and after birth in the overall DS-R score and the core, animal reminder, and contamination subscales. T1-T3 corresponds to trimester numbers, with T4 being the measurement after birth. The significant differences of disgust sensitivity between periods, results of post hoc tests, are shown with *p < 0.05, **p < 0.01 and ***p < 0.001. The graphs show the means and standard errors.

Post hoc tests showed that in the overall DS-R questionnaire, women scored significantly higher (had higher disgust sensitivity) in T2 (t_240_ = − 2.21, *p* = 0.028, Cohen’s d = − 0.29) and T4 (t_241_ = − 2.45, *p* = 0.015, Cohen’s d = − 0.32) than in T1. For the animal reminder subscale, women also scored significantly higher in T2 (t_240_ = − 2.42, *p* = 0.016, Cohen’s d = − 0.31) and in T4 (t_241_ = − 3.84, *p* < 0.001, Cohen’s d = − 0.50) than in T1. Additionally, women in T4 scored significantly higher than in T3 (t_241_ = − 2.63, *p* = 0.009, Cohen’s d = − 0.34) in this subscale. These results are illustrated in Fig. 2.

**Figure 3:**
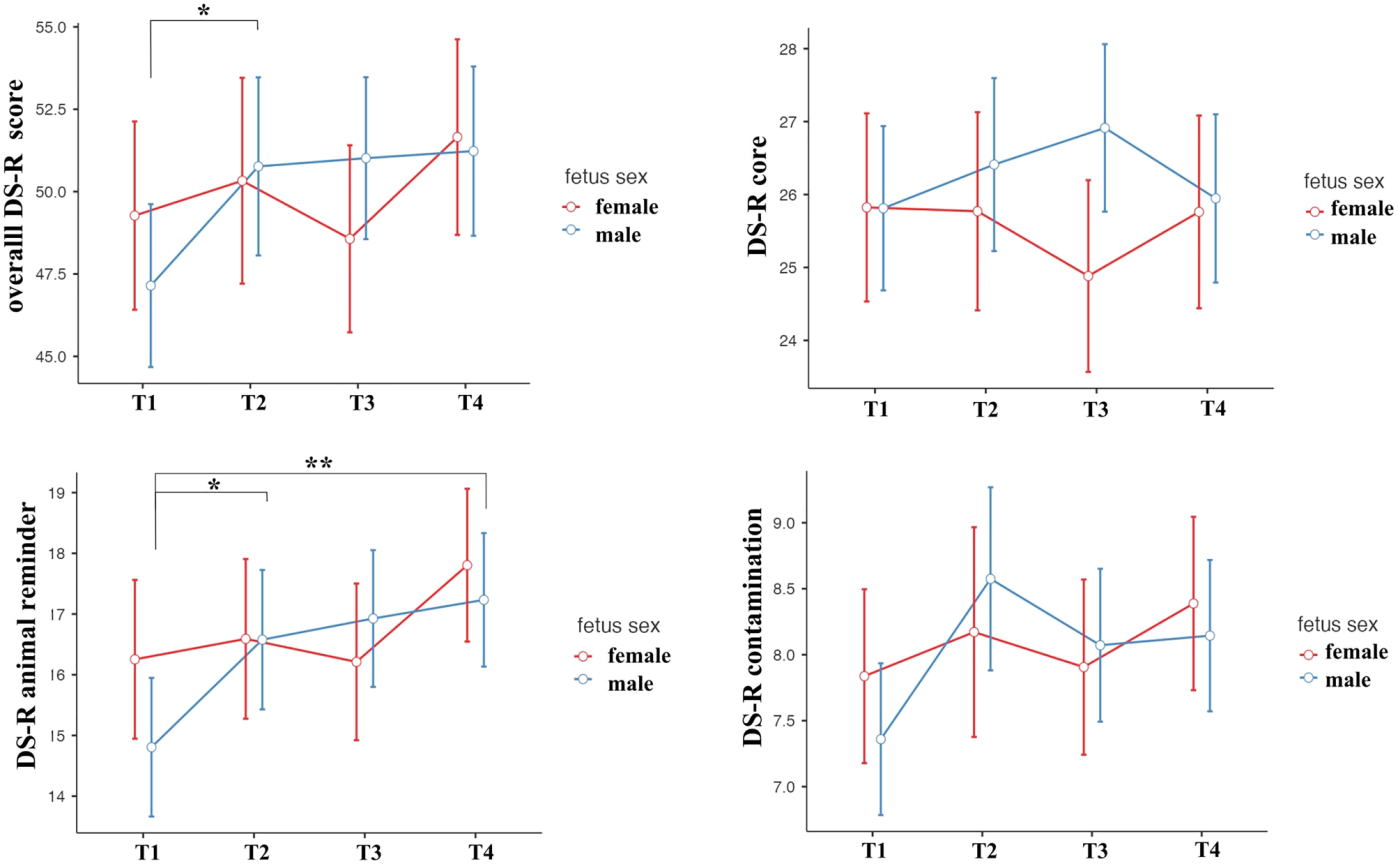
Longitudinal changes during pregnancy and after birth in the overall DS-R score, the core, animal reminder, and contamination subscales, separately for women pregnant with male and female fetus. T1-T3 corresponds to trimester numbers, with T4 being the measurement after birth. The significant differences of disgust sensitivity between periods, results of post hoc tests with the Bonferroni correction, are shown with *p < 0.05 and **p < 0.01. The graphs show the means and standard errors.

While no significant effect of fetus sex, nor of fetus sex in interaction with the pregnancy period was observed, post hoc tests were done to obtain results comparable with the replicated study. However, since there was no significant effect of the fetus sex in the first place, these post hoc tests were performed with the Bonferroni correction. They were done separately for each sex. These analyses showed that in the overall DS-R questionnaire, women pregnant with a male fetus scored significantly higher in T2 (t_134_ = − 2.71, *p*_*bonferroni*_ = 0.045, Cohen’s d = − 0.47) in comparison with T1. In the animal reminder subscale, women pregnant with a male fetus scored significantly higher in T2 (t_134_ = − 3.0, *p*_*bonferroni*_ = 0.020, Cohen’s d = − 0.52) and T4 (t_134_ = − 3.66, *p*_*bonferroni*_ = 0.002, Cohen’s d = − 0.63) when compared with T1. These results are illustrated in Fig. 3.

### The effect of recent health problems on disgust sensitivity

In the 1st trimester (T1), 24% of women reported having recent health problems. In the 2nd trimester (T2), 21% of women, and in the 3rd trimester (T3), 25% of women reported having recent health problems.

The ANCOVA test, controlled for age and fetus sex, showed significant effects of recent health problems on disgust sensitivity in the 1st trimester of pregnancy, specifically in the subscales core disgust (F_1,83_ =  6.40, *p* = 0.013, η^2^ =  0.07) and contamination disgust (F_1,83_ =  5.64, *p* = 0.020, η^2^ = 0.06 ). In the core disgust subscale, women who had recent health problems scored higher (n = 22, Mean = 29.1, SE = 1.39) than women that did not report any problems (n = 70, Mean = 25.1, SE = 0.77). Women with recent health problems also scored higher in the contamination disgust subscale (n = 22, Mean = 9.33, SE = 0.74) than women without health problems (n = 70, Mean = 7.29, SE = 0.41). No significant results were found in the animal reminder subscale. Similar effects were not observed in the later trimesters of pregnancy. However, a long-term effect of health problems in the first trimester on disgust sensitivity in the third trimester has been observed; women who reported recent health problems in T1 scored higher in the contamination subscale in T3 (n = 21, Mean = 9.09, SE = 0.70) than women who did not report any problems in T1 (n = 64, Mean = 7.43, SE = 0.41). This long-term effect of health problems in the first trimester on disgust sensitivity in the third trimester has not been observed in other subscales or in the overall DS-R score. No other long-term effect of health problems in the first (or second/third) trimester on disgust sensitivity in later trimesters (or in the postpartum period) was observed. The complete results of the ANCOVA tests are summarized in Table 2.

**Table 2:** ANCOVA tests showing the effect of recent health problems in each trimester on disgust sensitivity in different trimesters and after birth.

		Recent health problems (yes/no)											
		T1				T2				T3			
		df	F	*p*	η^2^	df	F	*p*	η^2^	df	F	*p*	η^2^
T1	Overall DS-R score	1,83	3.18	0.078	0.04		–	–	–		–	–	–
	Core		6.40	**0.013**	0.07		–	–	–		–	–	–
	Animal reminder		0.06	0.809	0.00		–	–	–		–	–	–
	Contamination		5.64	**0.020**	0.06		–	–	–		–	–	–
T2	Overall DS-R score	1,76	1.94	0.168	0.03	1,76	0.48	0.490	0.01		–	–	–
	Core		2.73	0.103	0.03		0.74	0.394	0.01		–	–	–
	Animal reminder		0.34	0.560	0.00		0.01	0.913	0.00		–	–	–
	Contamination		2.20	0.142	0.03		0.99	0.324	0.01		–	–	–
T3	Overall DS-R score	1,80	3.34	0.071	0.04	1,73	0.19	0.669	0.00	1,80	0.06	0.808	0.00
	Core		2.84	0.096	0.03		0.66	0.419	0.01		0.02	0.904	0.00
	Animal reminder		1.41	0.239	0.02		0.05	0.826	0.00		0.35	0.559	0.00
	Contamination		4.04	**0.048**	0.05		0.39	0.533	0.01		0.13	0.724	0.00
T4	Overall DS-R score	1,67	1.09	0.301	0.02	1,61	0.00	0.975	0.00	1,65	0.02	0.891	0.00
	Core		0.79	0.379	0.01		0.11	0.743	0.00		0.01	0.907	0.00
	Animal reminder		0.65	0.423	0.01		0.59	0.444	0.01		0.02	0.895	0.00
	Contamination		1.31	0.257	0.02		0.48	0.491	0.01		0.01	0.919	0.00

Controlled for age and fetus sex.

Significant values are in [bold].

### The effect of recent health problems on longitudinal changes in disgust sensitivity

Based on the observed significant effect of recent health problems on disgust sensitivity in the first trimester, the analysis using the linear mixed model was repeated, with the variable of recent health problems in each trimester now added as a covariate. This analysis included only the time of pregnancy (T1–T3) since the data regarding recent health problems has not been collected after birth.

In these new models, the longitudinal changes in disgust sensitivity changed, but remained significant for the animal reminder disgust subscale (F_2,162.8_ = 4.26, *p* = 0.016, R^2^ marginal = ﻿0.02) and in the overall DS-R score (F_2,162.7_ = 3.21, *p* = 0.043, R^2^ marginal = 0.02). The contamination (F_2,163.2_ = 2.17, *p* = 0.119, R^2^ marginal = 0.01) and core (F_2,163.5_ = 0.47, *p* = 0.628, R^2^ marginal = 0.03) disgust subscales were not significant.

The post hoc tests for this new model showed that women still scored higher in T2 in comparison with T1 in the overall DS-R (t_168_ = − 2.29, *p* = 0.023, Cohen’s d = − 0.35) and in the animal reminder subscale (t_168_ = − 2.60, *p* = 0.010, Cohen’s d = − 0.40).

Post hoc tests done separately for each sex (Bonferroni-corrected), in this new model showed that in the overall DS-R questionnaire, women pregnant with a male fetus scored significantly higher in T2 (t_94.6_ = − 2.84, *p*_*bonferroni*_ = 0.017, Cohen’s d = − 0.58) and T3 (t_94.5_ = − 2.71, *p*_*bonferroni*_ = 0.024, Cohen’s d = − 0.56) in comparison with T1. In the animal reminder subscale, women pregnant with a male fetus scored significantly higher in T2 (t_94.5_ = − 3.35, *p*_*bonferroni*_ = 0.003, Cohen’s d = − 0.69) and T3 (t_94.4_ = − 3.24, *p*_*bonferroni*_ =  0.005, Cohen’s d = − 0.67) when compared with T1.

## Discussion

Our main aim was to replicate the study by Żelaźniewicz and Pawłowski^30^, which focused on longitudinal changes in disgust sensitivity during pregnancy, and we also wished to explore whether recent health problems play a role in these changes. We observed significant changes in the overall DS-R score and in the animal reminder disgust subscale, but not in the core and contamination disgust subscales. Compared to the first trimester, we found that disgust sensitivity (both the overall DS-R score and the animal reminder disgust subscale) was higher in the second trimester and in the postpartum period. The results also showed that women who had experienced recent health problems reported higher disgust sensitivity in the first trimester on the core disgust subscale. After including these recent health problems into the main linear mixed model, longitudinal changes in disgust sensitivity during pregnancy remained significant for the animal reminder disgust subscale as well as the overall score. We also observed different trajectories of disgust sensitivity during pregnancy and after birth in women with male and female fetus.

### Longitudinal changes in disgust sensitivity

As in the original study^30^, our results showed significant longitudinal changes in disgust sensitivity. However, while the original study showed higher core disgust in the first trimester, which decreased throughout pregnancy and after birth, our study suggested a rather different pattern, with overall disgust and animal reminder disgust (but not core and contamination disgust) being lowest in the first trimester and increasing throughout pregnancy and after birth. Our results are therefore not consistent with the compensatory prophylaxis hypothesis (CPH) during pregnancy, as initially conceived and reflected in the studies of Fessler et al.^23^ and Żelaźniewicz and Pawłowski^30^, where disgust is expected to be highest in the first trimester. On the other hand, a core concept of the CPH is that compensatory changes in disgust sensitivity are underpinned by the immunosuppressive effects of progesterone^19–21^, and this could be supported by our results. Progesterone levels increase throughout pregnancy^33^ and the disgust sensitivity observed here follows the same trend. The only problematic part of this explanation would be the maintenance of high disgust sensitivity in the period after birth when progesterone levels decrease. This would suggest the existence of either a second mechanism that relates to elevated disgust sensitivity after birth, as a form of maternal behavioral immunity to protect the offspring, or that rising disgust in later pregnancy and post-partum can be jointly explained by a mechanism unrelated to progesterone.

Related to the individual subscales, longitudinal changes were significant only for the animal reminder subscale. This subscale is supposed to encompass stimuli that remind people of their animal nature and thus also their vulnerability and mortality^34^. The stimuli are all related to wounds, blood, mutilations and death. This subscale of the DS-R has been criticized previously^35^ for the unclear adaptive function of this form of disgust, specifically whether disgust is the most effective emotion for realizing our own mortality and why that would be beneficial. It seems that this subscale is quite different from the other two domains, which directly relate to pathogens and contagion. A series of studies by Kupfer^36^ showed that animal reminder disgust might be an empathic response to (for example) an injury rather than disgust in reaction to pathogen threat. The emotion is still described as disgust but stems more from the feelings of pain and horror, while pathogen disgust serves as a protection from infection. Therefore, the results of this subscale in this study could reflect different mechanisms occurring during pregnancy rather than simply a protection mechanism against pathogens. Considering that the animal reminder disgust is the highest before and after birth, it might be considered as an event closely linking humans with their animal nature, as well as a strong emotional experience. Women have been shown to experience higher fear of death in the third trimester^37^ and often experience fear of death during labor^38^. Anxiety symptoms have also been shown to be higher in the third trimester^39,40^. Anxiety and fear of birth experienced before birth has been shown to relate to postpartum anxiety^41^. Overall, it can be argued that animal reminder disgust, relating to injuries and death, could be increased before and after labor, because women at that time experience higher fear (of death) and anxiety in relation to childbirth.

The effect size (marginal R^2^ was 0.03, resp. 0.02) for the models focusing on longitudinal changes (overall disgust, resp. animal reminder disgust) was rather small. This shows that most of the differences in disgust sensitivity during pregnancy can be attributed to inter-individual differences, rather than changes within an individual. However, it should be considered that even small changes in disgust may be significant during pregnancy, which is a very sensitive period. This model could also quite possibly underestimate the actual size of the changes that occur, since the measuring tools—text questionnaires—are rather blunt instruments, even if effective ones. The observed effect size could therefore reflect only a fragment of larger changes happening.

### Effect of recent health problems on disgust sensitivity

In line with the CPH^4^, our results showed a significant effect of recent health problems in the first trimester on the core disgust subscale. Furthermore, while our results are the first to show this effect in pregnant women, they are consistent with previous research where higher disgust sensitivity was observed during the luteal phase in women who had experienced a recent infection^10^, in people who had been more frequently sick^11^, or in those who considered themselves more vulnerable to disease^12^. Related to the individual subscales, recent health problems did not influence animal reminder disgust. However, since animal reminder disgust is defined as a subscale relating to the need for humans to distance themselves from their animal nature, which reminds them of their vulnerability and mortality^34^, it does not relate to pathogen risk as much as the core subscale. Thus, it does not work as a mechanism to prevent infection and accordingly does not relate to the CPH. Therefore, we would not even expect to observe changes in this subscale when looking at a potential effect of recent health problems.

The fact that the effect of recent health problems was observed only in the first trimester supports the theory of the adaptive function of disgust. The first trimester is well known as a time of embryogenesis, when many developmental processes sensitive to disruption take place and therefore the mother and embryo need increased protection^42,43^. A similar adaptive function has been shown in regard to nausea and vomiting in early pregnancy^44–46^.

Based on these findings, the variable of recent health problems was added into the linear mixed model of longitudinal changes in disgust sensitivity. After this addition, the models with the overall DS-R score and the animal reminder subscale remained significant. Overall, it seems that longitudinal changes in disgust sensitivity are influenced by multiple factors, and differently so in each area of disgust. On one hand, the changes could be influenced by maternal health factors; on the other, they might be underpinned by physiological and related psychological changes accompanying pregnancy and the early postpartum period.

### Longitudinal changes in disgust sensitivity depending on the fetus sex

Another finding of this study, pertaining to the replicated study^30^, is the effect of fetus sex on longitudinal changes in disgust. These results are mixed, however, and support the findings of the replicated study only weakly. Our results showed that there was a near significant difference (*p* = 0.075) in longitudinal changes in overall disgust sensitivity between women pregnant with male and female fetuses. In women pregnant with a male fetus, overall disgust sensitivity and animal reminder disgust were significantly higher in the second trimester compared to the first trimester, with the animal reminder disgust additionally being significantly higher after birth in comparison with the first trimester. Overall, the specific results differed from the replicated study^30^. It seems that fetus sex might play some role in affecting disgust sensitivity during pregnancy, but the results are mixed.

The observed trend of increasing disgust sensitivity in mothers with a male fetus, along with the small effect (*p* = 0.084, Cohen’s d = − 0.38) of fetus sex on core disgust in the third trimester (these results are reported in the Supplement section), could however be explained in a similar fashion to the replicated study. Studies have shown that testosterone levels of mothers pregnant with male fetuses increased throughout pregnancy^28^, as did cortisol levels in the second half of pregnancy until the 30th week^27^. These hormones have been shown to have immunosuppressive effects^25,26^ with possible mediative relationships on behavior^47^. Therefore, increased disgust sensitivity could be playing a protective role as a result of potential immunosuppression. It needs to be said, however, that these results were observed mostly in the animal reminder subscale, so they might have entirely different underlying mechanisms, as discussed in section "Longitudinal changes in disgust sensitivity". Overall, these results do not bring strong support to the idea of fetus sex playing a role in affecting disgust sensitivity during pregnancy.

### Limitations

One of the main limitations of this study might be our use of the Disgust Scale-Revised (DS-R). We chose to use the DS-R in this study because it was used in the study that we aimed to replicate. However, this questionnaire has been critiqued several times for its unclear subscales^35,48^ or low internal reliability, especially regarding the contamination subscale^49,50^. In this study, we have also shown low internal reliability of the contamination subscale, for which we have therefore reported results but not discussed them. It needs to be mentioned that previous studies, such as the replicated study^30^ or other studies^21^ focused on disgust related to women’s reproductive health often do not report the reliability results for the questionnaire subscales or for the whole questionnaire at all. This could indicate that perhaps other studies have encountered the same problems with this subscale, even though they report and discuss these results.

In further research, then, it might be better to use the Three Domain Disgust Scale (TDDS)^35^ or visual questionnaires. The possibility of using behavioral tests, such as with tactile^51,52^ or olfactory stimuli^53^, which are also used to measure disgust sensitivity, should also be explored. However, special caution would be advised since pregnant women could have unexpected strong reactions (e.g. vomiting, faintness) to such tests, which could be ethically problematic in some cases.

On the other hand, it should be noted that some of the clearest results in our study were found in the animal reminder subscale, which is unique to the DS-R. Without it, we may have been unable to recognize significant changes occurring during pregnancy in this area of disgust.

## Conclusion

Our study showed that, in the first trimester, disgust sensitivity is increased in circumstances where there is a need for higher behavioral prophylaxis, such as when the mother has been recently sick. This could signal an insufficiently activated immune system. These findings are in line with the CPH. However, the longitudinal changes in disgust sensitivity seem to be more complex than the CPH can predict. Disgust sensitivity seems to be influenced not only by physiological changes, but also psychological changes that occur during pregnancy and after birth. The sex of the fetus also seems to play some role in affecting disgust sensitivity during pregnancy, but the results are mixed and should be explored further in the future to be able to come to a clearer conclusion.

## Supplementary Information

Supplementary Information.

## Data Availability

The raw data for this study are available at https://doi.org/10.6084/m9.figshare.19354688.v4.
