# Supplementary material for: Longitudinal changes in disgust sensitivity during pregnancy and the early postpartum period, and the role of recent health problems
Source: Sci Rep. 2023 Mar 23;13:4752. doi: 10.1038/s41598-023-31060-6 (PMC10036647; doi:10.1038/s41598-023-31060-6)
Supplement: Supplementary file 1 — Supplementary Information. [file 41598_2023_31060_MOESM1_ESM.docx]

**Supplementary material**

**Table S1.** The coding structure of the binary variable “health problems”

| **Yes = 1** | **No = 0** |
| --- | --- |
| cold | no problems |
| sore throat | allergies/pollen allergies |
| bronchititis | migraines |
| fever | hip pain |
| cough | nausea |
| tonsilitis | spotting |
| nasopharyngitis | vaginal discomfort |
| pneumonia | panic attacks |
| yersenia infection | headache |
| bladder infection | vaginal discharge |
| sinusitis | limb cramps |
|  | dysuria (related to pregnancy) |
|  | back pain (related to pregnancy) |
|  | constipation |
|  | reflux |
|  | tromboflebitis |
|  | rib pain |
|  | pregnancy diabetes (n = 1, in 3^rd^ trimester)* |
|  |  |

*Only health problems which were considered as an acute infection were coded as a yes = 1, while chronic problems, such as allergies or problems related to pregnancy (nausea, back pain) were coded as a no = 0*

** after excluding this woman from the analyses, the significance of the results did not change*

**Table S2**. The mean and standard deviation for every category of the DS-R in each measuring period, along with the Cronbach`s α value of internal consistency

|  | 1^st^ trimester | 2^nd^ trimester | 3^rd^ trimester | After birth |
| --- | --- | --- | --- | --- |
| DS-R | n = 92 | n = 81 | n = 86 | n = 72 |
| Overall score  Cronbach’s α | 50.6 (SD = 14.1)  0.832 | 52.2 (SD = 15.1) 0.869 | 51.8 (SD = 13.9)  0.862 | 51.9 (SD = 13.9)  0.832 |
| Core  Cronbach’s α | 26.3 (SD = 6.46)  0.581 | 26.6 (SD = 6.92)  0.688 | 26.7 (SD = 6.54)  0.696 | 26.0 (SD = 6.48)  0.660 |
| Animal reminder  Cronbach’s α | 16.5 (SD = 6.36)  0.744 | 17.5 (SD = 6.68)  0.809 | 17.3 (SD = 6.39)  0.805 | 17.7 (SD = 6.23)  0.718 |
| Contamination  Cronbach’s α | 7.85 (SD = 3.37)  0.546 | 8.14 (SD = 3.79)  0.648 | 7.81 (SD = 3.32)  0.570 | 8.24 (SD = 3.33)  0.570 |

**Table S3.** Intercorrelations of the DS-R subscales for each trimester and the postpartum period

| T1/T2/T3/T4 | Core | Animal reminder |
| --- | --- | --- |
| Animal reminder | 0.667*/ 0.687*/0.617*/0.686* |  |
| Contamination | 0.537*/0.552*/0.555*/0.601* | 0.602*/0.539*/0.511*/0.515* |

*The r values of Pearson correlation performed between all DS-R subscales, always within one time period (T1-T4; three trimesters and the postpartum period), the test significance is marked *p<0.001*

**Table** **S4.** Linear regression/t-test (or Mann-Whitney) models testing the effect of age/parity and fetus sex on disgust sensitivity. The n for the periods T1-T4 is 92, 81, 86 and 72 respectively.

|  | | age | | | | | parity | | | fetus sex | | | gestation age | | |
| --- | --- | --- | --- | --- | --- | --- | --- | --- | --- | --- | --- | --- | --- | --- | --- |
| DS-R | | t | B | Beta | *p* | R^2^ | t/U* | *p* | d/r*_rb_** | t | *p* | d | Tau B | *p* | d |
| T1 | Overall score | -1.52 | -0.50 | 0.33 | 0.133 | 0.025 | 1025* | 0.875 | 0.020* | 0.07 | 0.942 | 0.016 | 0.08 | 0.275 | 0.266 |
|  | Core | -2.28 | -0.34 | 0.15 | **0.025** | 0.055 | 0.85 | 0.399 | 0.178 | -0.50 | 0.621 | -0.107 | 0.13 | 0.101 | 0.406 |
|  | Animal reminder | -0.80 | -0.12 | 0.15 | 0.427 | 0.007 | 0.18 | 0.857 | 0.038 | 0.33 | 0.742 | 0.071 | 0.05 | 0.480 | 0.173 |
|  | Contamination | -0.51 | -0.04 | 0.08 | 0.611 | 0.003 | 0.17 | 0.865 | 0.036 | 0.64 | 0.527 | 0.137 | 0.01 | 0.876 | 0.038 |
| T2 | Overall score | -0.37 | -0.14 | 0.37 | 0.713 | 0.002 | 0.14 | 0.892 | 0.031 | -0.60 | 0.552 | -0.134 | -0.12 | 0.116 | -0.390 |
|  | Core | -0.25 | -0.04 | 0.17 | 0.796 | <0.001 | -0.03 | 0.979 | -0.006 | -0.91 | 0.364 | -0.205 | -0.12 | 0.121 | -0.390 |
|  | Animal reminder | -0.57 | -0.09 | 0.16 | 0.572 | 0.004 | 0.14 | 0.887 | 0.032 | -0.31 | 0.757 | -0.070 | -0.10 | 0.201 | -0.323 |
|  | Contamination | <0.01 | <0.01 | 0.09 | 0.998 | <0.001 | 0.34 | 0.736 | 0.076 | -0.16 | 0.872 | -0.036 | -0.10 | 0.226 | -0.310 |
| T3 | Overall score | -0.07 | -0.02 | 0.34 | 0.943 | <0.001 | 0.12 | 0.902 | 0.027 | -1.28 | 0.204 | -0.279 | -0.11 | 0.166 | -0.336 |
|  | Core | -0.21 | -0.03 | 0.16 | 0.833 | <0.001 | 0.46 | 0.648 | 0.099 | -1.75 | 0.084 | -0.381 | -0.13 | 0.079 | -0.429 |
|  | Animal reminder | -0.52 | -0.08 | 0.16 | 0.607 | 0.003 | 0.12 | 0.905 | 0.026 | -0.94 | 0.349 | -0.205 | -0.10 | 0.192 | -0.320 |
|  | Contamination | 1.15 | 0.09 | 0.08 | 0.253 | 0.016 | -0.61 | 0.542 | -0.132 | -0.14 | 0.891 | -0.030 | -0.01 | 0.946 | -0.016 |
| T4 | Overall score | -0.17 | -0.07 | 0.40 | 0.865 | <0.001 | 0.09 | 0.930 | 0.021 | 0.13 | 0.898 | 0.031 | 0.12 | 0.158 | 0.390 |
|  | Core | -0.80 | -0.15 | 0.18 | 0.427 | 0.009 | 0.02 | 0.982 | 0.005 | -0.02 | 0.981 | -0.006 | 0.13 | 0.126 | 0.429 |
|  | Animal reminder | 0.05 | 0.01 | 0.18 | 0.957 | <0.001 | 0.30 | 0.768 | 0.070 | 0.11 | 0.915 | 0.025 | 0.10 | 0.252 | 0.320 |
|  | Contamination | 0.75 | 0.07 | 0.09 | 0.456 | 0.008 | -0.23 | 0.822 | -0.054 | 0.39 | 0.701 | 0.091 | 0.03 | 9.736 | 0.086 |

*The number of participants (n) in the first trimester is 88 in the analysis of fetus sex, due to missing data and 84 in the analysis of gestation age for the same reason. In the period after birth, in the analysis of gestation age, the n is 66, also due to missing data. For testing the effect of parity on overall DS-R in T1, the homogeneity of variances was not met, therefore the Mann-Whitney test was used. The statistic value U and the value of rank biserial correlation for effect size are marked *. The effect size for t-tests is reported through the Cohen d value.*
